# Supplementary material for: Value of Artificial Intelligence in Evaluating Lymph Node Metastases
Source: Cancers (Basel). 2023 Apr 26;15(9):2491. doi: 10.3390/cancers15092491 (PMC10177013; doi:10.3390/cancers15092491)
Supplement: Supplementary file 1 [file cancers-15-02491-s001.zip › cancers-2346680-supplementary.pdf]

Supplementary material

Table S1. Search strategy for electronic databases.

Database	Search key, Search strategy of the 6th August 2022.
Pubmed	
	#1 "image"[Title/Abstract] AND "analysis"[Title/Abstract]<br>#2 "artificial"[Title/Abstract] AND "intelligence"[Title/Abstract]<br>#3 "morphometry"[Title/Abstract] OR "morphometric"[Title/Abstract] OR "histomorphometric"[Title/Abstract] OR "AI"[Title/Abstract] OR "algorithm*"[Title/Abstract] OR "neural network"[Title/Abstract] OR "neural networks"[Title/Abstract] OR "convolutional"[Title/Abstract] OR "deep-learning"[Title/Abstract] OR "deep-learning"[Title/Abstract] OR "computational"[Title/Abstract] OR "computerized"[Title/Abstract] OR "automated"[Title/Abstract] OR "machine-learning"[Title/Abstract] OR "machine-learning"[Title/Abstract]<br>#4 #1 OR #2 OR #3<br>#5 "metastases"[Title/Abstract] OR "metastasis"[Title/Abstract] OR "metastatic"[Title/Abstract]<br>#6 "lymphnode"[Title/Abstract] OR "lymphnodes"[Title/Abstract] OR "lymph node"[Title/Abstract] OR "lymph nodes"[Title/Abstract] OR "nodal"[Title/Abstract]<br>#7 #4 AND #5 AND #6<br>#8
Embase	
	#1 ('image'/exp OR image) AND ('analysis'/exp OR analysis)<br>#2 artificial AND ('intelligence'/exp OR intelligence)<br>#3 'morphometry'/exp OR morphometry OR morphometric OR histomorphometric OR ai OR algorithm* OR 'neural network'/exp OR 'neural network' OR 'neural networks'/exp OR 'neural networks' OR convolutional OR 'deep learning'/exp OR 'deep learning' OR computational OR computerized OR automated OR 'machine learning'/exp OR 'machine learning'<br>#4 #1 OR #2 OR #3<br>#5 'metastases'/exp OR metastases OR 'metastasis'/exp OR metastasis OR metastatic<br>#6 lymphnode OR 'lymphnodes'/exp OR lymphnodes OR 'lymph node'/exp OR 'lymph node' OR 'lymph nodes'/exp OR 'lymph nodes' OR nodal<br>#7 #4 AND #5 AND #6<br>#8 #7 AND [embase]/lim NOT ([embase]/lim AND [medline]/lim) AND ('article'/it OR 'article in press'/it OR 'conference paper'/it OR 'editorial'/it OR 'review'/it) AND (2000:py OR 2001:py OR 2002:py OR 2003:py OR 2004:py OR 2005:py OR 2006:py OR 2007:py OR 2008:py OR 2009:py OR 2010:py OR 2011:py OR 2012:py OR 2013:py OR 2014:py OR 2015:py OR 2016:py OR 2017:py OR 2018:py OR 2019:py OR 2020:py OR 2021:py OR 2022:py)
